# *rac*-(*rel*-1*R*,2*R*,4*S*)-Spiro­[bicyclo­[2.2.1]heptane-2,3′-indol]-2′-amine

**DOI:** 10.1107/S1600536811001048

**Published:** 2011-01-15

**Authors:** Andreas Lemmerer, Joseph P. Michael

**Affiliations:** a Molecular Sciences Institute, School of Chemistry, University of the Witwatersrand, Johannesburg, PO Wits 2050, South Africa

## Abstract

In the racemic title compound, C_14_H_16_N_2_, the aromatic ring component of the amino­indoline system occupies the *endo* cavity of the norbornane component. The aromatic ring lies at an angle of 74.12 (5)° to the plane defined by the four C atoms that comprises the rigid part of the boat-shaped six-membered ring of the norbornane unit. Pairs of mol­ecules assemble in the crystal structure, forming centrosymmetric hydrogen-bonded dimers *via* pairs of N—H⋯N hydrogen bonds through the *syn* H atom of the amine group.

## Related literature

For the synthesis, see: Fleming *et al.* (1986 ▶). For related compounds, see: Lemmerer & Michael (2010 ▶).
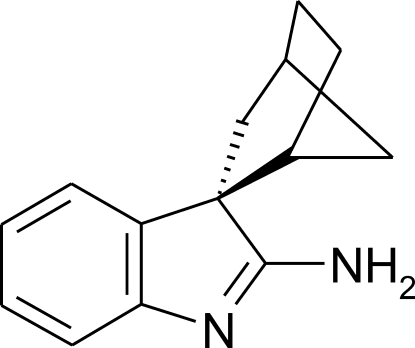


## Experimental

### 

#### Crystal data

C_14_H_16_N_2_
*M* *_r_* = 212.29
Orthorhombic, 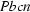

*a* = 19.2145 (14) Å
*b* = 11.3371 (8) Å
*c* = 10.3399 (7) Å
*V* = 2252.4 (3) Å^3^
*Z* = 8
Mo *K*α radiation
μ = 0.08 mm^−1^
*T* = 173 K
0.4 × 0.12 × 0.08 mm

#### Data collection

Bruker SMART 1K CCD area-detector diffractometer
Absorption correction: integration (*XPREP*; Bruker, 1999 ▶) *T*_min_ = 0.980, *T*_max_ = 0.994
16382 measured reflections
2728 independent reflections
1842 reflections with *I* > 2σ(*I*)
*R*_int_ = 0.078

#### Refinement

*R*[*F* ^2^ > 2σ(*F* ^2^)] = 0.046
*wR*(*F* ^2^) = 0.118
*S* = 1.04
2728 reflections
151 parameters
H atoms treated by a mixture of independent and constrained refinement
Δρ_max_ = 0.22 e Å^−3^
Δρ_min_ = −0.20 e Å^−3^

### 

Data collection: *SMART-NT* (Bruker, 1998 ▶); cell refinement: *SAINT* (Bruker, 1999 ▶); data reduction: *SAINT*; program(s) used to solve structure: *SHELXS97* (Sheldrick, 2008 ▶); program(s) used to refine structure: *SHELXL97* (Sheldrick, 2008 ▶); molecular graphics: *ORTEP-3 for Windows* (Farrugia, 1997 ▶) and *DIAMOND* (Brandenburg, 1999 ▶); software used to prepare material for publication: *WinGX* (Farrugia, 1999 ▶) and *PLATON* (Spek, 2009 ▶).

## Supplementary Material

Crystal structure: contains datablocks global, I. DOI: 10.1107/S1600536811001048/ng5096sup1.cif

Structure factors: contains datablocks I. DOI: 10.1107/S1600536811001048/ng5096Isup2.hkl

Additional supplementary materials:  crystallographic information; 3D view; checkCIF report

## Figures and Tables

**Table 1:** Hydrogen-bond geometry (Å, °)

*D*—H⋯*A*	*D*—H	H⋯*A*	*D*⋯*A*	*D*—H⋯*A*
N2—H1⋯N1^i^	0.927 (19)	2.10 (2)	3.0112 (18)	169 (2)

Symmetry code: (i) 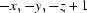.

**Fig. 1.**
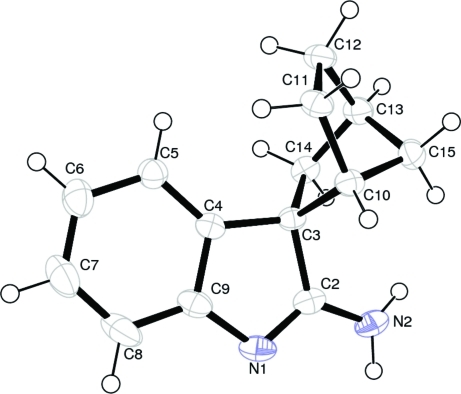
The asymmetric unit of (I) showing the atomic numbering scheme. Displacement ellipsoids are shown at the 50% probability level.

**Fig. 2.**
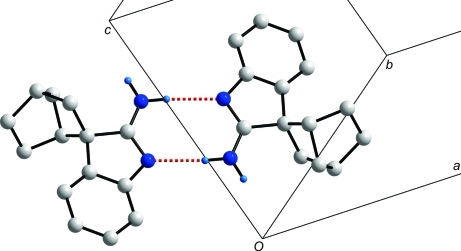
Packing diagram of (I). Intermolecular N—H···N hydrogen bonds are shown as dashed red lines forming dimers. Note that the anti H2 is not used in any hydrogen bonding interactions.

## supplementary crystallographic information

### Comment

The racemic compound (*rac*)-(*rel*-1*R*,2*R*,4*S*)-Spiro[bicyclo[2.2.1] heptane-2,3'-indol]-2'-amine (I) is an intermediate in the synthesis of a model oxindole prepared during the development of methodology aimed at the total synthesis of the complex spiro-oxindole alkaloid gelsemine (Fleming *et al.*, 1986). The solid state packing of the title compound involves forming centrosymmetric hydrogen-bonded pairs of molecules, generated by interactions from the *syn* H1 of the amine to the N1 lone pair of the oxindole backbone (See Fig 2). Formation of dimeric pairs is seen in related oxindole compounds (Lemmerer & Michael, 2010). The *anti* H of the amine group is not involved in any hydrogen bonding interactions.

### Experimental

The compound was prepared as described previously (Fleming *et al.*, 1986). Crystals of (I) were grown by slow evaporation at ambient conditions of a hexane–chloroform solution (1:1 *v*/*v*).

### Refinement

The C-bound H atoms were geometrically placed (C—H bond lengths of 0.95 (aromatic CH), 0.99 (methylene CH_2_) and 1.00 (methine CH) Å) and refined as riding with *U*_iso_(H) = 1.2*U*_eq_(C). The N-bound H atoms were located in the difference map and coordinates refined freely, with *U*_iso_(H) = 1.5*U*_eq_(N).

### Crystal data

**Table:**

C_14_H_16_N_2_	*F*(000) = 912
*M**_r_* = 212.29	*D*_x_ = 1.252 Mg m^−^^3^
Orthorhombic, *P**b**c**n*	Mo *K*α radiation, λ = 0.71073 Å
Hall symbol: -P 2n 2ab	Cell parameters from 876 reflections
*a* = 19.2145 (14) Å	θ = 2.9–25.5°
*b* = 11.3371 (8) Å	µ = 0.08 mm^−^^1^
*c* = 10.3399 (7) Å	*T* = 173 K
*V* = 2252.4 (3) Å^3^	Needle, colourless
*Z* = 8	0.4 × 0.12 × 0.08 mm

### Data collection

**Table:**

Bruker SMART 1K CCD area-detector diffractometer	2728 independent reflections
Radiation source: sealed tube	1842 reflections with *I* > 2σ(*I*)
graphite	*R*_int_ = 0.078
ω scans	θ_max_ = 28.0°, θ_min_ = 2.1°
Absorption correction: integration (*XPREP*; Bruker, 1999)	*h* = −22→25
*T*_min_ = 0.980, *T*_max_ = 0.994	*k* = −14→14
16382 measured reflections	*l* = −13→13

### Refinement

**Table:**

Refinement on *F*^2^	0 restraints
Least-squares matrix: full	H atoms treated by a mixture of independent and constrained refinement
*R*[*F*^2^ > 2σ(*F*^2^)] = 0.046	*w* = 1/[σ^2^(*F*_o_^2^) + (0.0594*P*)^2^ + 0.0604*P*] where *P* = (*F*_o_^2^ + 2*F*_c_^2^)/3
*wR*(*F*^2^) = 0.118	(Δ/σ)_max_ = 0.005
*S* = 1.04	Δρ_max_ = 0.22 e Å^−^^3^
2728 reflections	Δρ_min_ = −0.20 e Å^−^^3^
151 parameters	

### Special details

**Table:**

Experimental. Numerical integration absorption corrections based on indexed crystal faces were applied using the *XPREP* routine (Bruker, 1999)
Geometry. All e.s.d.'s (except the e.s.d. in the dihedral angle between two l.s. planes) are estimated using the full covariance matrix. The cell e.s.d.'s are taken into account individually in the estimation of e.s.d.'s in distances, angles and torsion angles; correlations between e.s.d.'s in cell parameters are only used when they are defined by crystal symmetry. An approximate (isotropic) treatment of cell e.s.d.'s is used for estimating e.s.d.'s involving l.s. planes.

### Fractional atomic coordinates and isotropic or equivalent isotropic displacement parameters (Å^2^)

**Table:**

	*x*	*y*	*z*	*U*_iso_*/*U*_eq_	
C2	0.08033 (7)	0.08297 (13)	0.39207 (14)	0.0269 (3)	
C3	0.12640 (7)	0.16703 (11)	0.31300 (13)	0.0221 (3)	
C4	0.11622 (7)	0.27794 (12)	0.39302 (13)	0.0239 (3)	
C5	0.13484 (8)	0.39471 (13)	0.37348 (14)	0.0299 (3)	
H5	0.16	0.417	0.2983	0.036*	
C6	0.11627 (8)	0.47945 (14)	0.46537 (16)	0.0394 (4)	
H6	0.1289	0.5597	0.4527	0.047*	
C7	0.07970 (9)	0.44686 (16)	0.57446 (17)	0.0484 (5)	
H7	0.0676	0.5051	0.6366	0.058*	
C8	0.06029 (9)	0.33061 (16)	0.59495 (18)	0.0481 (5)	
H8	0.0356	0.3086	0.6708	0.058*	
C9	0.07762 (7)	0.24683 (14)	0.50236 (14)	0.0311 (3)	
C10	0.20345 (7)	0.11810 (12)	0.30821 (13)	0.0250 (3)	
H10	0.2183	0.0743	0.3874	0.03*	
C11	0.25299 (7)	0.21793 (13)	0.26686 (14)	0.0287 (3)	
H11A	0.3022	0.1927	0.2727	0.034*	
H11B	0.2462	0.2892	0.3208	0.034*	
C12	0.23141 (8)	0.24110 (13)	0.12412 (14)	0.0302 (3)	
H12A	0.211	0.3208	0.114	0.036*	
H12B	0.2718	0.2333	0.0652	0.036*	
C13	0.17676 (7)	0.14452 (12)	0.09730 (14)	0.0286 (3)	
H13	0.1715	0.1232	0.004	0.034*	
C14	0.10853 (7)	0.17663 (12)	0.16523 (13)	0.0263 (3)	
H14A	0.071	0.1209	0.1414	0.032*	
H14B	0.0938	0.2577	0.1426	0.032*	
C15	0.20324 (8)	0.04395 (12)	0.18327 (14)	0.0312 (4)	
H15A	0.2503	0.0163	0.1584	0.037*	
H15B	0.1705	−0.0234	0.1873	0.037*	
N1	0.05733 (6)	0.12635 (12)	0.50174 (12)	0.0341 (3)	
N2	0.06590 (7)	−0.02770 (11)	0.35451 (14)	0.0334 (3)	
H1	0.0310 (10)	−0.0677 (15)	0.3982 (17)	0.05*	
H2	0.0746 (9)	−0.0498 (16)	0.2725 (18)	0.05*	

### Atomic displacement parameters (Å^2^)

**Table:**

	*U*^11^	*U*^22^	*U*^33^	*U*^12^	*U*^13^	*U*^23^
C2	0.0181 (6)	0.0322 (8)	0.0303 (8)	−0.0025 (6)	−0.0042 (6)	0.0085 (6)
C3	0.0195 (6)	0.0246 (7)	0.0223 (7)	−0.0020 (5)	−0.0009 (5)	0.0028 (5)
C4	0.0185 (6)	0.0306 (7)	0.0226 (7)	−0.0003 (5)	−0.0014 (5)	0.0012 (6)
C5	0.0304 (8)	0.0306 (7)	0.0287 (8)	−0.0010 (6)	0.0003 (6)	−0.0016 (6)
C6	0.0383 (9)	0.0367 (9)	0.0432 (10)	−0.0013 (7)	−0.0016 (7)	−0.0111 (7)
C7	0.0422 (10)	0.0553 (11)	0.0476 (11)	−0.0032 (8)	0.0076 (8)	−0.0249 (9)
C8	0.0388 (10)	0.0679 (12)	0.0375 (10)	−0.0101 (9)	0.0170 (8)	−0.0137 (9)
C9	0.0223 (7)	0.0430 (8)	0.0278 (8)	−0.0042 (6)	0.0026 (6)	−0.0007 (6)
C10	0.0197 (6)	0.0281 (7)	0.0272 (8)	0.0004 (6)	−0.0008 (6)	0.0045 (6)
C11	0.0222 (7)	0.0357 (8)	0.0282 (8)	−0.0035 (6)	0.0017 (6)	−0.0012 (6)
C12	0.0306 (8)	0.0347 (8)	0.0254 (8)	−0.0075 (6)	0.0058 (6)	−0.0014 (6)
C13	0.0330 (8)	0.0318 (8)	0.0211 (7)	−0.0055 (6)	0.0002 (6)	−0.0038 (6)
C14	0.0271 (7)	0.0274 (7)	0.0243 (7)	−0.0032 (6)	−0.0048 (6)	0.0015 (6)
C15	0.0269 (7)	0.0273 (7)	0.0394 (9)	0.0012 (6)	0.0052 (7)	−0.0028 (6)
N1	0.0267 (6)	0.0434 (7)	0.0321 (7)	−0.0086 (6)	0.0057 (5)	0.0052 (6)
N2	0.0302 (7)	0.0309 (7)	0.0390 (8)	−0.0092 (5)	0.0000 (6)	0.0082 (6)

### Geometric parameters (Å, °)

**Table:**

C2—N1	1.3126 (19)	C10—C15	1.5413 (19)
C2—N2	1.3424 (19)	C10—H10	1
C2—C3	1.5362 (18)	C11—C12	1.555 (2)
C3—C4	1.5178 (19)	C11—H11A	0.99
C3—C14	1.5698 (18)	C11—H11B	0.99
C3—C10	1.5819 (19)	C12—C13	1.5423 (19)
C4—C5	1.386 (2)	C12—H12A	0.99
C4—C9	1.3974 (19)	C12—H12B	0.99
C5—C6	1.398 (2)	C13—C14	1.531 (2)
C5—H5	0.95	C13—C15	1.533 (2)
C6—C7	1.379 (2)	C13—H13	1
C6—H6	0.95	C14—H14A	0.99
C7—C8	1.386 (2)	C14—H14B	0.99
C7—H7	0.95	C15—H15A	0.99
C8—C9	1.389 (2)	C15—H15B	0.99
C8—H8	0.95	N2—H1	0.927 (19)
C9—N1	1.420 (2)	N2—H2	0.900 (18)
C10—C11	1.5394 (19)		
			
N1—C2—N2	122.04 (13)	C10—C11—H11A	111.2
N1—C2—C3	114.92 (12)	C12—C11—H11A	111.2
N2—C2—C3	123.02 (13)	C10—C11—H11B	111.2
C4—C3—C2	98.60 (11)	C12—C11—H11B	111.2
C4—C3—C14	116.42 (11)	H11A—C11—H11B	109.1
C2—C3—C14	115.78 (11)	C13—C12—C11	103.43 (11)
C4—C3—C10	115.35 (10)	C13—C12—H12A	111.1
C2—C3—C10	109.78 (10)	C11—C12—H12A	111.1
C14—C3—C10	101.45 (10)	C13—C12—H12B	111.1
C5—C4—C9	119.74 (13)	C11—C12—H12B	111.1
C5—C4—C3	132.72 (12)	H12A—C12—H12B	109
C9—C4—C3	107.47 (12)	C14—C13—C15	101.24 (11)
C4—C5—C6	119.44 (14)	C14—C13—C12	109.37 (11)
C4—C5—H5	120.3	C15—C13—C12	101.41 (12)
C6—C5—H5	120.3	C14—C13—H13	114.4
C7—C6—C5	120.13 (15)	C15—C13—H13	114.4
C7—C6—H6	119.9	C12—C13—H13	114.4
C5—C6—H6	119.9	C13—C14—C3	104.05 (11)
C6—C7—C8	121.12 (15)	C13—C14—H14A	110.9
C6—C7—H7	119.4	C3—C14—H14A	110.9
C8—C7—H7	119.4	C13—C14—H14B	110.9
C7—C8—C9	118.71 (16)	C3—C14—H14B	110.9
C7—C8—H8	120.6	H14A—C14—H14B	109
C9—C8—H8	120.6	C13—C15—C10	94.67 (10)
C8—C9—C4	120.81 (14)	C13—C15—H15A	112.8
C8—C9—N1	126.50 (14)	C10—C15—H15A	112.8
C4—C9—N1	112.63 (13)	C13—C15—H15B	112.8
C11—C10—C15	99.78 (11)	C10—C15—H15B	112.8
C11—C10—C3	109.24 (11)	H15A—C15—H15B	110.3
C15—C10—C3	102.41 (10)	C2—N1—C9	105.77 (11)
C11—C10—H10	114.6	C2—N2—H1	117.8 (11)
C15—C10—H10	114.6	C2—N2—H2	119.7 (12)
C3—C10—H10	114.6	H1—N2—H2	117.2 (16)
C10—C11—C12	102.88 (11)		
			
N1—C2—C3—C4	−8.15 (14)	C2—C3—C10—C11	−162.78 (11)
N2—C2—C3—C4	173.58 (13)	C14—C3—C10—C11	74.23 (13)
N1—C2—C3—C14	−133.10 (13)	C4—C3—C10—C15	−157.66 (11)
N2—C2—C3—C14	48.64 (18)	C2—C3—C10—C15	92.08 (12)
N1—C2—C3—C10	112.82 (13)	C14—C3—C10—C15	−30.90 (12)
N2—C2—C3—C10	−65.45 (16)	C15—C10—C11—C12	39.45 (13)
C2—C3—C4—C5	−170.93 (15)	C3—C10—C11—C12	−67.48 (13)
C14—C3—C4—C5	−46.4 (2)	C10—C11—C12—C13	−4.84 (14)
C10—C3—C4—C5	72.31 (19)	C11—C12—C13—C14	74.58 (14)
C2—C3—C4—C9	6.05 (13)	C11—C12—C13—C15	−31.79 (14)
C14—C3—C4—C9	130.55 (12)	C15—C13—C14—C3	39.32 (13)
C10—C3—C4—C9	−110.72 (13)	C12—C13—C14—C3	−67.16 (14)
C9—C4—C5—C6	1.7 (2)	C4—C3—C14—C13	121.15 (12)
C3—C4—C5—C6	178.34 (14)	C2—C3—C14—C13	−123.67 (12)
C4—C5—C6—C7	0.0 (2)	C10—C3—C14—C13	−4.90 (13)
C5—C6—C7—C8	−0.4 (3)	C14—C13—C15—C10	−57.33 (12)
C6—C7—C8—C9	−0.8 (3)	C12—C13—C15—C10	55.32 (12)
C7—C8—C9—C4	2.5 (3)	C11—C10—C15—C13	−58.27 (12)
C7—C8—C9—N1	−174.52 (15)	C3—C10—C15—C13	54.09 (12)
C5—C4—C9—C8	−2.9 (2)	N2—C2—N1—C9	−174.97 (13)
C3—C4—C9—C8	179.63 (14)	C3—C2—N1—C9	6.75 (16)
C5—C4—C9—N1	174.45 (12)	C8—C9—N1—C2	174.96 (16)
C3—C4—C9—N1	−2.99 (16)	C4—C9—N1—C2	−2.23 (16)
C4—C3—C10—C11	−52.53 (15)		

### Hydrogen-bond geometry (Å, °)

**Table:**

*D*—H···*A*	*D*—H	H···*A*	*D*···*A*	*D*—H···*A*
N2—H1···N1^i^	0.927 (19)	2.10 (2)	3.0112 (18)	169 (2)

Symmetry codes: (i) −*x*, −*y*, −*z*+1.
